# Effects of nitrogen top-dressing treatments and development stages on morphological, colorimetric and biochemical characteristics of fresh bean pods (*Phaseolus vulgaris* L.)

**DOI:** 10.1038/s41598-026-57239-1

**Published:** 2026-06-07

**Authors:** Aykut Şener

**Affiliations:** Faculty of Agriculture, Department of Field Crops, Isparta University of Applied Sciences, 32200 Isparta, Turkey

**Keywords:** Bean, Development stages, Top-dressing, Pod quality, Biochemical, Biochemistry, Physiology, Plant sciences

## Abstract

Nitrogen management and harvest timing are critical determinants of fresh bean (*Phaseolus vulgaris* L.) pod quality; however, their combined effects on morphological, colorimetric and biochemical characteristics during pod development remain insufficiently characterized. This study evaluated the effects of four nitrogen top-dressing fertilizers (ammonium sulfate [AS], slow-release fertilizer [SRF], nitropower [NP], and urea) and five pod development stages (BBCH 72–81) on pod morphology, color parameters, antioxidant capacity, phenolic compounds, flavonoids, and protein content under field conditions. Nitrogen top-dressing significantly prolonged days to 50% flowering and pod setting times, with the latest flowering observed under urea treatment compared to basal fertilization. Pod length and width increased with advancing development stages, while pod water content declined from early stages to physiological maturity. Colorimetric analysis revealed a marked increase in lightness (L*), yellowness (b*) and chroma values with maturation, accompanied by a decrease in hue angle, indicating reduction in greenness and increase in yellowness. Antioxidant capacity exhibited a stage-dependent pattern, with lower values at intermediate stages and higher values at late development stages, particularly under urea treatment. Total phenolic, flavonoid and protein content increased significantly at the final development stage. Strong positive correlations were detected between antioxidant capacity, phenolic, flavonoid and protein content, whereas pod water content showed significant negative correlations with these quality traits. Overall, the results demonstrate that development stage plays a key role in determining pod quality, while nitrogen source influences the magnitude of these responses. Late harvest stages (BBCH 79–81), together with stable nitrogen sources such as SRF and AS, could be employed to improve nutritional and antioxidant properties of fresh bean pods.

**Supplementary Information:**

The online version contains supplementary material available at 10.1038/s41598-026-57239-1.

## Introduction

The legume family (Fabaceae) contains approximately 730 genera and 19,400 species, making it the third largest among the flowering plants^1^. Leguminous species play critical roles in enhancing soil fertility and sustainable agricultural practices due to their capacity for nitrogen fixation, as well as their significance in human and animal nutrition. *Phaseolus vulgaris* L., originated from Mesoamerica and the Andes and despite its origin, it is a significant species and is cultivated in many countries throughout the world today. The global production of dry beans and fresh beans were 28.5 and 29.8 million tons in 2023, respectively^2^.

The fresh pods and dried seeds of the beans contain essential nutrients, such as proteins, amino acids, complex carbohydrates, dietary fibers and oligosaccharides, as well as beneficial phytochemicals including phenolic compounds, saponins, alkaloids and tannins. Consequently, *P. vulgaris* has garnered attention as a functional and nutraceutical food in contemporary times^3,4^. Moreover, its low carbon footprint renders the bean plant a significant component of sustainable nutrition systems^3^.

Fresh bean pods start growing rapidly shortly after blooming, exhibiting significant variations in morphological and biochemical characteristics dependent on development stages. For fresh consumption, pods are harvested when pod elongation is generally completed and grain filling rate increased. However, genotype, climate, soil conditions, cultivation techniques, stress factors alter growth and nutrient content, which could have significant effects on consumption characteristics (taste, texture, and aroma). Marketing strategies are also often shaped by the pod’s development stage and nutritional composition.

To achieve a high yield and quality in bean production, the soil nitrogen levels must be sufficient. It is desirable for soils to be rich in nitrogen, especially for the synthesis of proteins and nucleic acids. Although symbiotic nitrogen fixation occurs with *Rhizobium*, biotic and abiotic stresses could constrain nitrogen fixation. Nitrogen requirements of bean plants vary according to their development stage, and that top-dressing treatment during the vegetative phase influences yield and pod composition^5–9^.

In basal fertilization (BF), diammonium phosphate (DAP, 18–46%) with a high phosphorus content, is widely used, phosphorus application enhances root development, nodulation, and nitrogen fixation capacity. Early development can positively affect the plant’s nitrogen-fixing capacity and photosynthetic efficiency. Phosphate containing fertilizers significantly improve plant growth, plant height, pod number, and total yield of beans^10–12^. In top-dressing, different nitrogen sources, such as ammonium sulfate (AS) [(NH_4_)_2_SO_4_], nitro power (NP) (33% N, 24% SO_3_) and urea [(NH_2_)_2_CO] (46% N) improve vegetative growth, leaf area, biomass, and chlorophyll content, leading to higher photosynthetic efficiency. Previous studies have shown that nitrogen in the form of ammonium facilitates nutrient uptake by lowering soil pH; and together with sulfur, it promotes amino acid (cysteine and methionine) synthesis, protein metabolism and antioxidant defense systems^13–16^. Urea provides rapid nitrogen supply during the early growth phases of plant development. When used at sufficient levels, it positively affects plant height, root and leaf size, as well as yield and yield components. However, applying excessive doses of urea could suppress symbiotic nitrogen fixation, root development and reduce photosynthetic activity^14,17^.

In recent years, slow-release fertilizers (SRF) have gained increasing importance in agricultural production. SRFs reduce nutrient losses by releasing plant nutrients in a controlled manner (over several weeks), ensuring availability of plant nutrients for longer periods. The release rate of plant nutrients in SRFs varies in soils due to temperature, humidity, pH, and microbial activity^18,19^. According to the Association of American Plant Food Control Offices^20^ nutrients remained available within the soil for a long time after SRF treatment. However, studies investigating the responses of different development stages of fresh beans to SRF and other nitrogen fertilizer types are quite limited.

The study was designed to reflect practical fertilization strategies commonly used by growers, where basal fertilization with DAP is often applied alone, while bacterial inoculation and additional nitrogen top-dressing are used selectively depending on production goals and input costs. Therefore, the present study aimed to evaluate whether basal fertilization alone is sufficient and to determine the effects of ammonium sulfate, slow-release fertilizer, nitro power, and urea treatments on the morphological and phytochemical characteristics of green beans harvested at different development and maturation stages for fresh consumption.

## Materials and methods

### Plant material and experimental design

The KON16 bean line, which has white seeds with a cuboid seed type (oblongus), dwarf growth, low pod stringiness and high yield potential, was developed through selection from local population for its stable agronomic performance^21,22^. Seed production, cultivation processes, and fertilizer treatments were carried out at the research farms of Isparta University of Applied Sciences (37° 45′ N and 30° 33′ E, 997 m). The research area exhibits climatic characteristics of a transitional zone between coastal and continental climate zones.

**Table 1:** Monthly average temperature and precipitation values for 2024 growing season and long-term averages (1929–2024).

Climatic Factors	Years	April	May	June	July	August	September	Avg./Total
Average Temperature (°C)	2024	16.8	17.1	27.1	26.6	26.5	16.8	22.6
	1929–2024	10.9	15.5	20.0	23.5	23.4	18.9	18.7
Precipitation (mm)	2024	25.2	74.1	7.4	36.7	4.0	27.2	174.6
	1929–2024	51.1	56.7	35.4	15.7	13.9	18.7	191.5

*: Data were obtained from the Turkish State Meteorological Service (MGM).

The mean temperature during April–September period was 22.6 °C in 2024, which was considerably higher than the long-term average of 18.7 °C. Monthly comparisons indicated that temperatures in April, May, June, July, and August were consistently higher than the long-term values, with the greatest deviations observed in April and June. In contrast, September exhibited slightly lower temperatures than the long-term average. Total precipitation between April to September was 174.6 mm, which was slightly lower than the long-term of 191.5 mm for the same period. However, the distribution of rainfall varied throughout the months. Precipitation was higher than average in May and July, whereas June and August received substantially lower rainfall compared to long-term values. (Table 1).

Overall, the 2024 growing season was characterized by higher temperatures and lower total precipitation compared to the long-term averages, indicating a warmer and relatively drier climatic pattern.

The experimental soil was classified as silty loam with a slightly alkaline reaction (pH 7.7) and non-saline conditions (EC = 0.248 dS m^− 1^). The high CaCO_3_ content (27%) indicated a calcareous soil. The soil had low organic matter (1.76%), moderate available phosphorus (12.2 mg kg^− 1^), high potassium (936 mg kg^− 1^) and calcium (18200 mg kg^− 1^), and sufficient magnesium (1413 mg kg^− 1^).

The experiment was conducted in randomized complete block design with split plots and three replications. Nitrogen top-dressing treatments were assigned to the main plots, while development stages (harvest times) were considered as subplots. Prior to planting, the land was prepared by plowing and disc harrowing. The experimental plots were 8 m^2^ (4 × 2 m), arranged with a row spacing of 50 cm and plant spacing of approximately 8 cm, with each plot consisting of 4 rows. All plots were treated with 18-46-0 DAP fertilizer at a concentration of 30 kg ha^− 1^ N and 60 kg ha^− 1^ P_2_O_5_ for basal fertilization.

Approximately 250–300 healthy seeds were counted for each plot and sown on April 26 2024 in hand-opened rows with 50 seeds per row. Top-dressing treatments were carried out when the plants reached BBCH code 21 (Principal growth stage 2: first side shoot visible)^23^. Nitrogen top-dressing was applied with four types of fertilizer: ammonium sulfate 21% N (AS), slow-release fertilizer 21% N (SRF), nitropower 33% N (NP), and urea 46% N. For all top-dressings, the amount of fertilizer to be applied was calculated separately to provide 100 kg ha^− 1^ of pure nitrogen. No fertilizer treatment other than basal fertilization was applied to the BF plots. This experimental design was established to reflect common farmer practices in fresh bean production systems. Growers commonly apply only basal fertilization with DAP at sowing to reduce input costs and generally do not apply bacterial inoculation or additional nitrogen top-dressing. Therefore, the objective of this study was not to compare fertilizer forms under equal total nitrogen levels, but to evaluate whether basal fertilization alone is sufficient and to determine which top-dressing nitrogen source may provide better agronomic and quality-related benefits under practical field conditions.

**Fig. 1:**
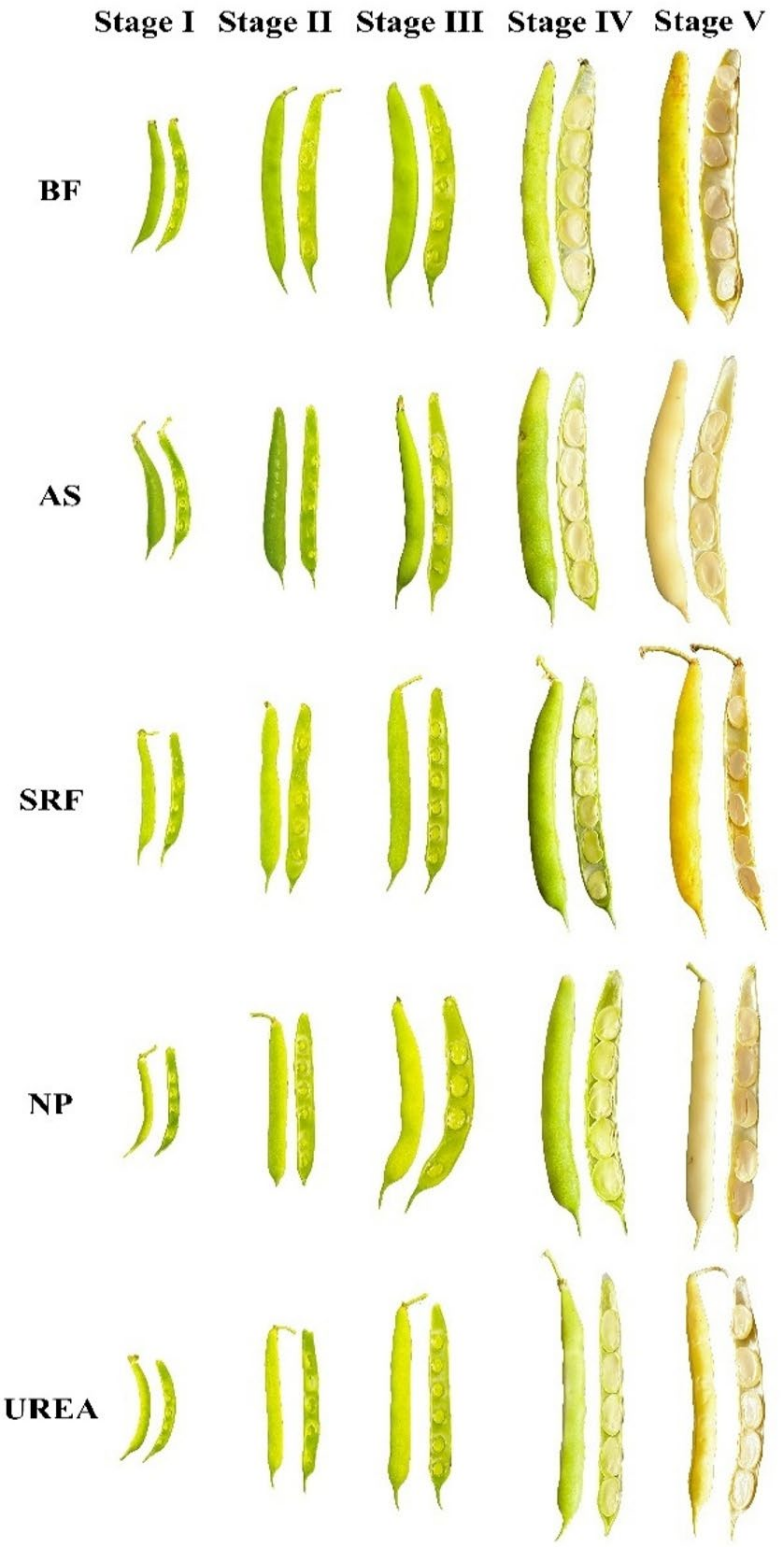
Pod development stages with top-dressing treatments. BF: basal fertilization; AS: ammonium sulfate; SRF: slow-release fertilizer; NP: nitropower; UREA: urea.

The development and maturation stages of the beans were determined based on the basic growth stages defined for beans in the BBCH Monograph, specifically for stages 7 and 8^23^. Pods were harvested at 5 different development stages: Stage 1: development of fruit code 72, Stage 2: development of fruit code 75, Stage 3: development of fruit code 77, Stage 4: development of fruit code 79, and Stage 5: ripening of fruit and seed code 81 (Fig. 1). Each development stage was considered as an independent harvest stage within the split-plot design and treated as a separate subplot factor rather than as repeated measurements from the same experimental unit. In this study, the term “vegetative period” refers to the pre-flowering stage, whereas “vegetation period” refers to the entire growth duration from sowing to harvest. Observations were made during vegetation periods throughout the field trials. Days to 50% flowering and 50% pod setting times were recorded when approximately half of the plants in each plot reached the respective stage. Vegetation period was determined as the number of days from sowing to harvest maturity. Depending on weather conditions and soil moisture, plants were irrigated with drip irrigation at intervals of 4–12 days. Weed control was carried out by hoeing.

### Morphological measurements and color parameters of pods

Fresh pods collected at each development stage were brought to the laboratory on the same day. In each plot, approximately 20–30 pods were randomly sampled at each development stage to represent biological replicates, with a higher number of pods collected at earlier stages due to smaller pod size. As part of the morphological measurements, pod length (cm) and pod width (mm) were measured using an electronic caliper and pod water content (%) was calculated based on the fresh weight after drying the samples in an oven at 80 ± 1 °C for 72 h.

Pod colorimetric parameters, including lightness (L*), redness (a*) and yellowness (b*) were measured using the Konica Minolta CR-400 colorimeter (Konica Minolta, Japan). For colorimetric analyses, measurements were performed on three randomly selected pods from each plot, with three readings taken at different points on each pod. Chroma (C*) (1) and hue angle (°) (2) values were calculated according to the following equations.

1 $$Metric{\text{ }}Chroma:\,C^{*} = \sqrt {\left( {a^{*} } \right)^{2} + (b^{*} )^{2} }$$

2 $$Metric{\text{ }}Hue - Angle:h = \tan ^{{ - 1}} \left( {\frac{{b^{*} }}{{a^{*} }}} \right)\left( {\deg ree} \right)$$

### Determination of phytochemical content of pods

Pods at each development stage were stored at -20 °C until Cupric Reducing Antioxidant Capacity (CUPRAC), 2,2-diphenyl-1-picrylhydrazyl (DPPH), total phenolic and flavonoid analyzes were performed. For extraction, pods were frozen in liquid nitrogen and subsequently ground into a fine powder using a mortar and pestle. Two grams of sample were weighed, and 20 mL of 80% methanol was added. The mixture was then incubated in an ultrasonic bath at 40 °C for 30 min and centrifuged at 10,000 rpm for 20 min. Antioxidant capacity (CUPRAC and DPPH), total phenolic and flavonoid content were determined from the resulting aliquots.

The CUPRAC test was based on the reduction of the colored copper(I)-neocuproine chelate^24^. The diluted extract (0.5 mL) was mixed with 1 mL of Cu(II) chloride (10 mM), 1 mL of neocuproine (7.5 mM), 1 mL of ammonium acetate buffer (1 M) and 0.6 mL of distilled water. Absorbance against a reagent blank was measured at 450 nm (Shimadzu UV-1280, Kyoto, Japan) after 30 min at room temperature. Results were expressed as Trolox equivalent per gram of fresh weight (µmol Trolox g^− 1^ FW).

The DPPH assay was performed based on the ability of antioxidants to reduce the DPPH radical^25^. The diluted extract (1 mL) was mixed with 1 mL of 99% ethanol and 2 mL of 0.2 mM DPPH solution. Ethanol was used as a blank and DPPH solution without sample as a control and measured at 515 nm after 30 min of dark incubation at room temperature. The DPPH radical-scavenging activity was expressed as Trolox equivalent (µmol Trolox g^− 1^ FW).

Total phenolic content (TPC) was determined using a modified Folin-Ciocalteau method^26^. Sample extracts (0.1 mL) were mixed with 2.5 mL of deionized water and 0.1 mL of Folin-Ciocalteu reagent. After 6 min, 0.5 mL of 20% sodium carbonate was added, and absorbance was measured at 760 nm using a spectrophotometer after 30 min. Total phenolic content was expressed as gallic acid equivalents (GAE) in mg g^− 1^ fresh weight (mg GAE g^− 1^ FW). Total phenolic content was calculated using a calibration curve prepared with gallic acid standard solutions at concentrations ranging from 20 to 100 µg mL^− 1^, with high linearity (R^2^ = 0.9968).

Total flavonoid content (TFC) was assessed via aluminum chloride reaction^27^. Sample extracts (0.25 mL) were mixed with deionized water (1.25 mL), and 75 µL of 5% sodium nitrite. After 6 min, 0.15 mL of 10% aluminum chloride was added, followed by a 5-min rest period. Then, 0.5 mL of 1 M sodium hydroxide solution and 0.275 mL distilled water were added for a final volume of 2.5 mL. Absorbance was measured at 510 nm after 15 min using a spectrophotometer. Flavonoid content was expressed as (±)-catechin equivalents in mg g^− 1^ fresh weight (mg catechin g^− 1^ FW). Total flavonoid content was calculated using a calibration curve prepared with (±)-catechin standard solutions at concentrations ranging from 25 to 400 µg mL^− 1^ (R^2^ = 0.9952).

For total protein analysis, 1 g of sample was extracted with 5 mL of 0.05 M cold Tris buffer (pH 8.0)^28^. The homogenates were centrifuged at 14,000 rpm for 20 min at 4 °C, and the total protein content was determined from the resulting supernatant according to the Bradford method^29^. The absorbance of the samples was determined at a wavelength of 595 nm, and the known concentrations of bovine serum albumin (BSA) were used to create the standard curve (120 to 600 µg mL^− 1^, R^2^ = 0.9977). Total protein content was expressed mg BSA g^− 1^ FW.

### Statistical analysis

All data were analyzed using a randomized complete block design with split-plots and three replications. Statistical analyses were performed using the TOTEMSTAT software (version 1.0) and differences between the means were compared using the TUKEY test (*p* ≤ 0.05)^30^. Additionally, Pearson linear correlation coefficients were calculated, and principal component analysis (PCA) was performed using OriginPro software (version 2025, OriginLab, Northampton, MA). PCA was conducted using standardized data based on the correlation matrix. The variables included in the analysis were pod length, pod width, pod water content, color parameters (L*, a*, b*, chroma, and hue angle), CUPRAC, DPPH, TPC, TFC and protein content. Principal components with eigenvalues greater than 1 (Kaiser criterion) were considered significant.

## Results and discussion

### Phenological and morphological observations

**Fig. 2:**
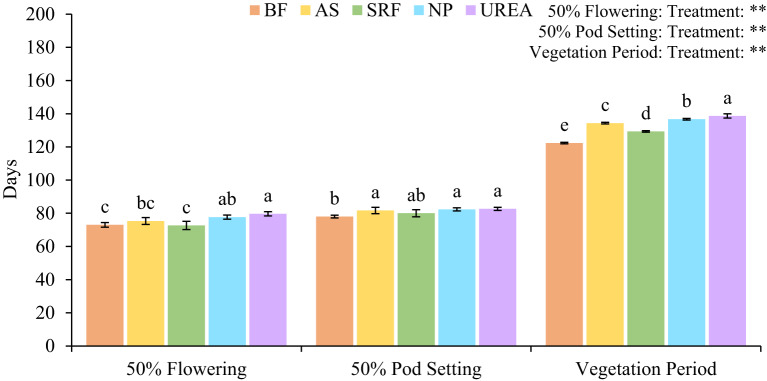
Mean values for 50% flowering times, 50% pod setting times and vegetation period of beans treated with different top-dressing fertilizers. BF: basal fertilization; AS: ammonium sulfate; SRF: slow-release fertilizer; NP: nitropower; UREA: urea.

Top-dressing treatments on bean plants had significant effects on phenological parameters including days to 50% flowering time, 50% pod setting time and vegetation period (Fig. 2; Supplementary Table S1). The earliest flowering occurred in SRF and BF treatments. The other nitrogen top-dressing treatments prolonged days to 50% flowering times, delaying the transition to the generative period. The latest flowering occurred in the plots treated with urea. All top-dressing treatments were within the same significance group, but days to 50% pod setting time was shorter in the BF treatment. Days to 50% pod setting times increased significantly with top-dressing nitrogen fertilizers, and the longest periods were recorded in urea, NP, AS and SRF treatments, respectively. In terms of vegetation period, a wide variation was observed among the top-dressing plots, and similar to days to 50% pod setting time, the shortest vegetation period was observed from BF treatment. Overall, the top-dressing fertilizer treatments significantly increased the vegetation period (Fig. 2). The current results are consistent with studies reporting that nitrogen treatment delays phenological processes, such as flowering, pod formation, and ripening in bean cultivation^31^.

Nitrogen is a limiting nutrient in plant growth, and its excessive application can delay the transition to the generative period by promoting vegetative growth^32,33^. Fertilizers containing rapidly soluble and highly available nitrogen, such as urea and NP, prolong vegetative growth period and delay transition from vegetative period to generative period in beans. However, prolonging vegetative growth under certain conditions has the potential to increase biomass accumulation and crop yield. Nitrogen availability plays an important role in regulating plant phenology, particularly flowering time, through its interaction with development and signaling pathways^34,35^. Under low nitrogen conditions, plants may accelerate flowering as a stress response, whereas sufficient nitrogen availability can delay the transition to the reproductive stage^36^.These responses are associated with nitrogen-mediated regulation of growth dynamics and development timing, ultimately affecting pod development and maturation processes. Similar nitrogen-dependent effects on phenological traits, particularly delayed flowering and maturity under nitrogen fertilization, have been reported in common bean under field conditions^14^.

**Fig. 3:**
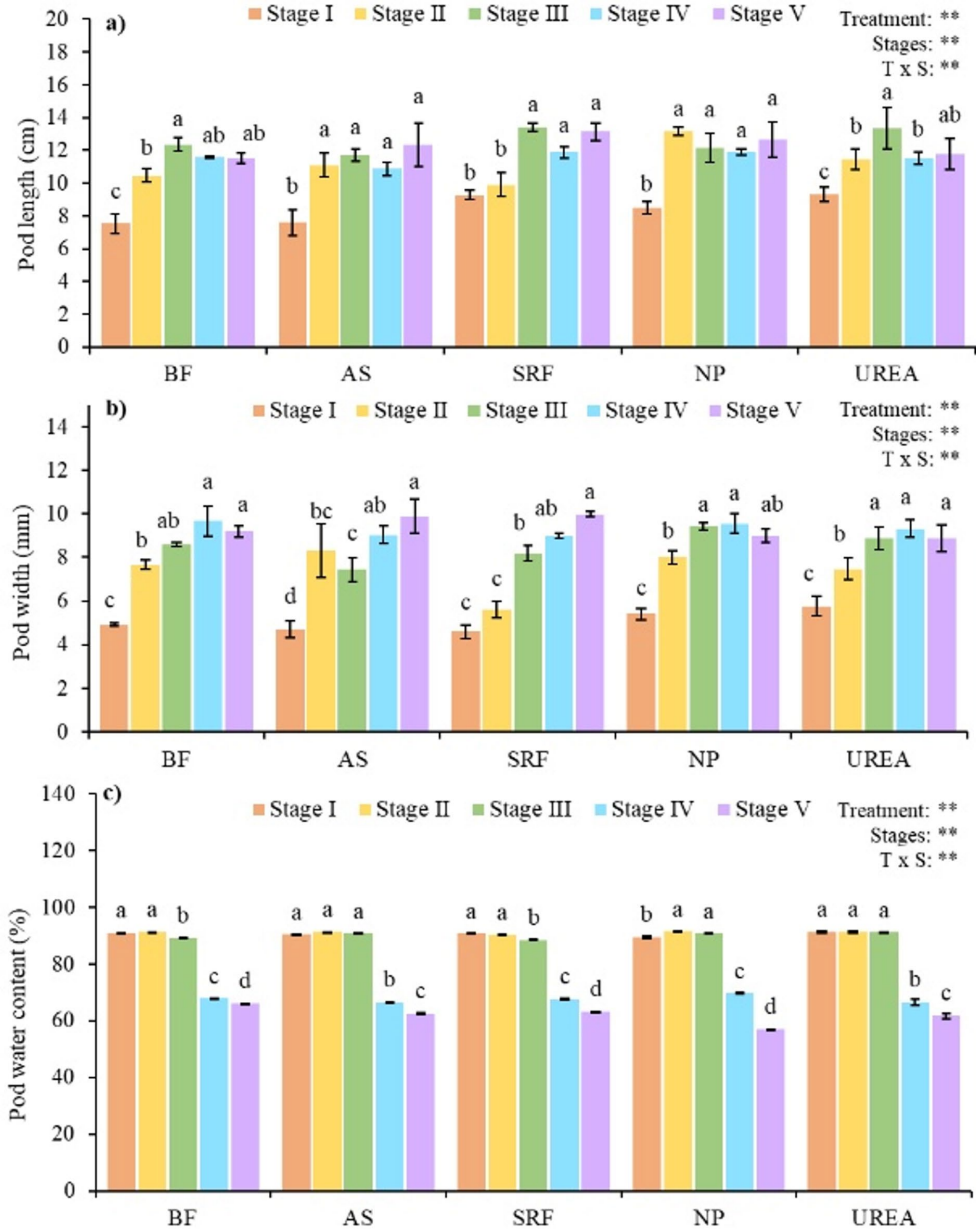
Morphological characteristics of bean pods harvested at different stages of bean plants treated with different nitrogen top-dressing treatments. BF: basal fertilization; AS: ammonium sulfate; SRF: slow-release fertilizer; NP: nitropower; UREA: urea.

The results of pod length, pod width, and pod water content, harvested at different periods after nitrogen top-dressing treatments, are shown in Fig. 3a. Pod length was significantly affected by both fertilizer treatments and development stages. The shortest pod lengths were obtained in the 1st development stage under both BF and top-dressing nitrogen treatments. Significant increases in pod length were observed until the 3rd development stage in all treatments except NP, while the longest average pod length was obtained in the 2nd development stage under NP treatment. Pod length decreased slightly after the 3rd period due to seed filling, ripening, and moisture reduction. When fertilizer types were compared, AS treatment did not show a significant difference from BF, whereas other top-dressing fertilizers increased pod length. Among these treatments, NP and SRF showed the most pronounced positive effects on pod length (Supplementary Table S2). These results indicate that nitrogen availability during early pod development promotes vegetative growth, cell division, and cell expansion, thereby contributing to pod elongation^37^. Similar positive effects of nitrogen fertilization on pod growth and yield have also been reported in beans^32,33^.

Pod width showed significant variation depending on both nitrogen top-dressing treatments and pod development stages (Fig. 3b). In both BF and top-dressing nitrogen treatments, pod width increased significantly with delayed harvest periods. The highest pod widths were generally observed at the 4th and 5th development stages, indicating that pod enlargement continued until seed filling and physiological maturation (Supplementary Table S2). In BF, NP, and urea treatments, the highest pod widths were recorded at the 4th stage, whereas in AS and SRF treatments, maximum values were observed at the 5th stage. With maturation, the transport of assimilates and nutrients from the plant to the developing seeds increases, resulting in larger seeds and consequently wider pods. Increased pod width is indirectly associated with yield and market quality. Higher nitrogen availability may enhance photosynthetic activity and carbohydrate production, thereby supporting pod filling and width enlargement^38^. Furthermore, nitrogen uptake continues until the pod filling stage, with peak absorption occurring between the beginning of pod formation and pod filling^39^. Continuous nitrogen availability throughout the development period positively contributes to pod filling and pod width.

Pod water content was significantly affected by both fertilizer treatments and development stages (Fig. 3c). When the average of fertilizer treatments across all development stages was compared, the highest pod water content was observed in BF treatment, followed by urea and AS treatments (Supplementary Table S2). In all treatments, the highest pod water content was measured during the early pod development, particularly at the 2nd development stage, while the lowest values were recorded at the 5th development stage, indicating a gradual decline during maturation. The observed decrease is mainly associated with seed filling and physiological ripening, during which assimilates and nutrients are increasingly transported from the plant to the developing seeds. As dry matter accumulation increases, pod moisture content decreases and physiological maturity is reached. Similar findings have been reported in beans where higher nitrogen availability increased fresh pod weight and delayed pod maturation^40^.

### Colorimetric analyses

Development stages and top-dressing treatments significantly affected the color properties of the fresh bean pods [lightness (L*), red-green (a*), yellow-blue (b*), chroma (C*), and hue angle (h°)]. Significant differences were found between development stages and treatments (*p* ≤ 0.05). As development progressed, the brightness of the pods increased, indicating that the pod color becomes lighter as pods mature^41^. The highest L* values were observed at the 5th development stage for all top-dressing treatments (Table 2). L* values are associated with sample brightness, where higher values indicate lighter color and lower values indicate darker tones^42,43^. Among the treatment averages, the lowest L* value was found in SRF treatment. The other treatments did not differ significantly in terms of L* values.

The a* values decreased from 1st to the 3rd development stages, indicating an increase in greenness, and reached their lowest values at the 3rd stage. Thereafter, a* values increased toward zero, indicating reduction in green coloration during maturation. At the 5th development stage, a* values were close to zero (-2.38), reflecting a substantial loss of greenness and the transition toward yellow coloration (Table 2). Similar color transitions during pod development were reported^43^. Compared to basal fertilization, all treatments were found to affect green color value of bean pods. Among the treatments across development stages, the lowest a* values were observed under urea, SRF, and NP treatments, respectively. Nitrogen deficiency has been reported to reduce chlorophyll content and leaf greenness^44^, which is consistent with the observed trends in this study. On the other hand, nitrogen fertilizers promote the growth of vegetative parts and chlorophyll synthesis, as was observed in the present study as well.

All fertilizer treatments increased b* values throughout pod development stages. The lowest b* value was observed in the 1st development stage, while the highest value was observed at the 5th development stage. Increased b* values indicate chlorophyll degradation during pod maturation, resulting in carotenoid accumulation. The AS, NP and urea treatments had higher b* values than BF and SRF treatments (Table 2). Nitrogen availability influences these processes by regulating chlorophyll synthesis and degradation dynamics, thereby affecting color development in bean pods. The biosynthesis of carotenoids and anthocyanins accompanies chlorophyll degradation throughout the plant development stages^45^. Green color in immature bean pods is related to chlorophyll concentration, which gradually decreases with maturation^46,47^.

**Table 2:** Colorimetric values of bean pods harvested at different stages of pod development subjected to different nitrogen top-dressing treatments.

Colorimetric Parameters	Treatment	Pod Development Stages					Avg.	Significance
		1	2	3	4	5		
L*	BF	34.12c	35.66c	33.74c	62.91b	70.49a	47.38a	Treatment: ** Stages: ** T x S: **
	AS	27.71d	34.64c	39.19c	59.27b	68.76a	45.91a	
	SRF	31.01d	38.02c	44.47bc	42.03b	61.89a	43.48b	
	NP	34.50c	43.21b	47.79b	43.15b	64.95a	46.72a	
	UREA	29.83d	41.98c	41.34c	58.73b	67.72a	47.92a	
	Avg.	31.43e	38.70d	41.31c	53.22b	66.76a	-	
a*	BF	-14.14c	-15.22c	-15.98c	-11.37b	-2.50a	-11.84a	Treatment: ** Stages: ** T x S: **
	AS	-13.51c	-15.41 cd	-17.06d	-11.39b	-4.10a	-12.29a	
	SRF	-12.69b	-16.61c	-18.59c	-18.60c	-1.98a	-13.70b	
	NP	-11.29b	-18.38c	-20.19c	-19.18c	-1.82a	-14.17b	
	UREA	-13.92b	-18.45c	-18.58c	-14.86b	-1.51a	-13.46b	
	Avg.	-13.11b	-16.82d	-18.08d	-15.08c	-2.38a	-	
b*	BF	16.48c	20.85c	21.00c	33.49b	40.42a	26.45b	Treatment: ** Stages: ** T x S: **
	AS	18.04d	21.37d	27.01c	35.41b	44.05a	29.18a	
	SRF	13.20d	22.80c	25.92bc	29.12ab	33.16a	24.84b	
	NP	14.25d	26.95c	32.53b	31.04bc	39.60a	28.88a	
	UREA	17.15c	25.63b	27.58b	39.49a	36.10a	29.19a	
	Avg.	15.82e	23.52d	26.81c	33.71b	38.67a	-	
Chroma	BF	21.71c	25.83c	26.39c	35.37b	40.51a	29.96b	Treatment: ** Stages: ** T x S: **
	AS	22.54d	26.35d	31.94c	37.20b	44.24a	32.45a	
	SRF	18.35c	28.21b	31.90ab	34.56a	33.22a	29.25b	
	NP	18.18c	32.62b	38.29a	36.49ab	39.64a	33.05a	
	UREA	22.10c	31.58b	33.25b	42.20a	36.13b	33.05a	
	Avg.	20.58d	28.92c	32.36b	37.16a	38.75a	-	
Hue Angle °	BF	130.64a	126.12b	127.27b	108.86c	93.50d	117.28b	Treatment: ** Stages: ** T x S: **
	AS	126.83a	125.79a	122.27b	107.83c	95.31d	115.61c	
	SRF	133.66a	126.11b	125.67bc	122.76c	93.53d	120.35a	
	NP	128.46a	124.30b	121.83b	121.74b	92.62c	117.79b	
	UREA	129.24a	125.78b	123.96b	110.62c	92.39d	116.40bc	
	Avg.	129.77a	125.62b	124.20b	114.36c	93.47d	-	

BF: basal fertilization; AS: ammonium sulfate; SRF: slow-release fertilizer; NP: nitropower; UREA: urea.

Means followed by different letters within the same row or column indicate significant differences according to Tukey’s test at *p* ≤ 0.05. ** indicates significance at *p* ≤  0.01.

Chroma values increased from the early to the late development stages. The lowest chroma values were determined in the 1st development stage in all treatments, and the highest values were determined in the 5th development stage. Color saturation increased with pod maturation, and chroma values showed a trend similar to b* values, with lower values observed under SRF and BF treatments and higher values under NP, urea, and AS treatments (Table 2). These results indicate that the availability of nutrients affects not only the pigment composition but also the visual color saturation, which is an important factor in terms of marketability and consumer preference^27^. Increased chroma value may also be related to antioxidant capacity^48^.

Hue angle values decreased with pod development, reflecting the gradual loss of green coloration and the increasing dominance of yellow tones during pod maturation. Hue angle is closely associated with chlorophyll retention and green color intensity, and its reduction indicates chlorophyll degradation and the progression of physiological maturity during pod development^49^. The highest hue angle value was determined in the 1st development stage, while the lowest value was observed in the 5th development stage. The decrease became more pronounced at the 4th and 5th development stages. Fertilizer treatments had also significant effects on hue angle values. The highest value was measured in SRF treatment, whereas the lowest value was observed in AS treatment (Table 2). Slower nitrogen release pattern of SRF may have delayed pod maturation and helped preservation of green color for a longer period. Similar to SRF treatment, a sharp decrease in hue angle value was observed in NP treatment from the 4th to the 5th development stage. It was reported that green bean pods are dark green during early pod formation and gradually become lighter green as development progresses^50^.

### Phytochemical components

**Fig. 4:**
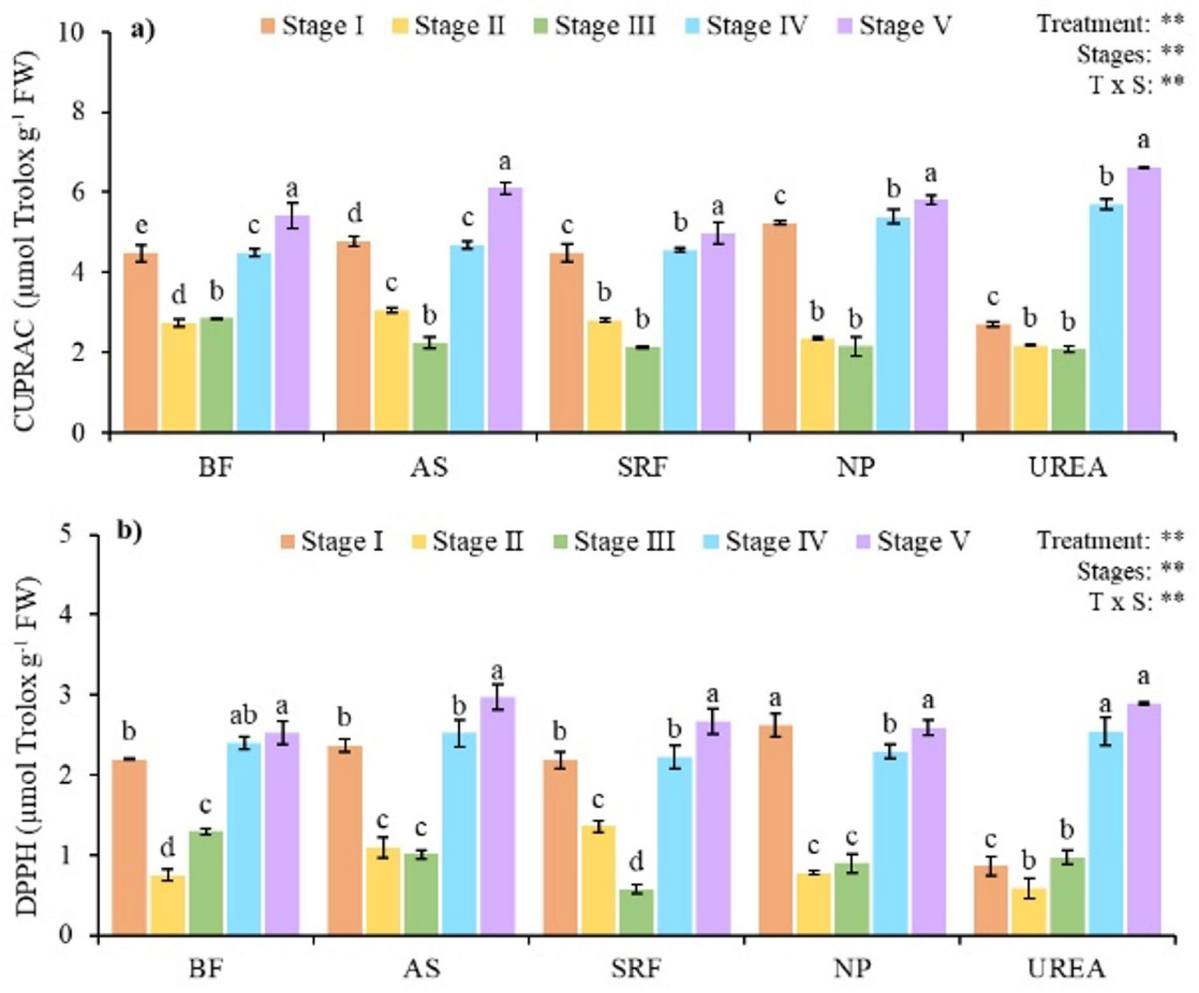
(**a**) CUPRAC and (**b**) DPPH activities of bean pods harvested at different stages and subjected to different nitrogen top-dressing treatments. BF: basal fertilization; AS: ammonium sulfate; SRF: slow-release fertilizer; NP: nitropower; UREA: urea; CUPRAC: cupric reducing antioxidant capacity; DPPH: 2,2-diphenyl-1-picrylhydrazyl radical scavenging activity.

The effects of different top-dressing treatments on the total antioxidant capacity, TPC, TFC and total protein content of bean pods harvested at different development stages were evaluated. Antioxidant capacity was determined using CUPRAC and DPPH methods, and the results showed that antioxidant capacity varied significantly depending on both development stage and fertilizer treatment (Fig. 4). CUPRAC analysis showed that antioxidant capacity increased significantly at the 4th and 5th development stages across all treatments, while the lowest values were observed at the 3rd stage (Fig. 4a). Among the treatments, the highest CUPRAC activity was recorded at the 5th stage with urea treatment, whereas the lowest activity was observed at the 3rd stage with SRF treatment (Supplementary Table S3). Antioxidant capacity decreased from 1st to 3rd development stage (except for BF), followed by a marked increase during seed filling and maturation that may be associated with metabolic changes occurring during pod development. In urea treatment, antioxidant capacity remained relatively low during the early development stages. This pattern may be associated with preferential nitrogen allocation to growth and pod development processes during the early stages of plant development rather than to the accumulation of antioxidant compounds. During the later stages, particularly pod filling and maturation, nitrogen availability may have contributed to drive protein synthesis, accumulation of phenolic compounds and flavonoids, resulting in higher antioxidant capacity. Since nitrogen is an essential component of amino acids, proteins, chlorophyll, and several secondary metabolites, it plays an important role in regulating plant metabolism and antioxidant compound biosynthesis^51^. Therefore, increased antioxidant capacity observed in late development stages suggests enhanced synthesis of these metabolites rather than a direct fertilizer effect. However, this should be interpreted as a possible physiological response rather than a direct conclusion.

DPPH results showed a similar trend to CUPRAC, with antioxidant activity decreasing from the 1st to the 3rd development stages, except for urea treatment. At the 4th stage, radical scavenging activity increased significantly and continued to increase at the 5th stage, except in BF and urea treatments. The lowest values were detected in the 3rd stage with SRF, and the highest in the 5th development stage with AS treatments (Fig. 4b). These results indicate that antioxidant activity decreased at early stages and increased with pod maturation and the results reflect the antioxidant potential of fresh bean pods, which have been reported to be a potential natural source of antioxidants^52^.

**Fig. 5:**
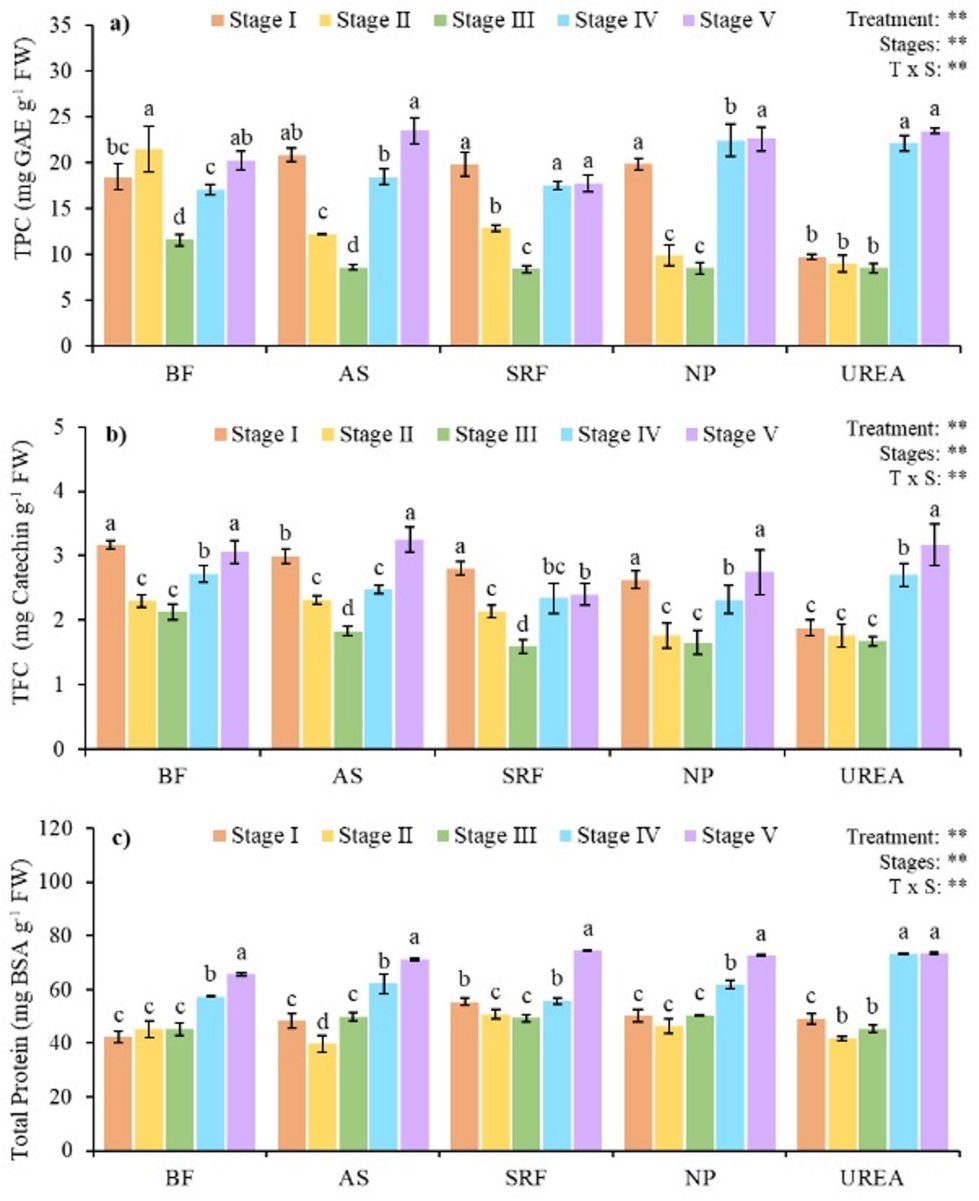
(**a**) Total phenolic content, (**b**) total flavonoid content, and (**c**) total protein content of bean pods harvested at different development stages with different nitrogen top-dressing treatments. BF: basal fertilization; AS: ammonium sulfate; SRF: slow-release fertilizer; NP: nitropower; UREA: urea; TPC: total phenolic content; TFC: total flavonoid content.

TPC showed a wide variation between 8.39 and 23.47 mg GAE g^− 1^ FW (Fig. 5a). Both top-dressing treatments and development stages significantly affected TPC of bean pods (Supplementary Table S3). Production, composition and accumulation of phenolic compounds can be affected by stress conditions^53^. Phenolic compounds have the potential to scavenge free radicals, activate redox enzymes, or act as metal chelators^54^, and these properties provide cells protection against oxidative damage^55,56^. Nitrogen status of plants influences the biosynthesis of phenolic compounds through its role in carbon-nitrogen balance and the regulation of the phenylpropanoid pathway, which is responsible for the production of many secondary metabolites^57,58^. Accumulation of total phenolics varied among the treatments; however, the lowest TPC was consistently observed at the 3rd development stage, while higher levels were generally detected toward the final stages, suggesting that pods harvested at later development stages, including seed filling and maturation, may exhibit enhanced phytochemical composition, as reflected by the higher TPC values observed at later development stages.

Flavonoids constitute an important group of bioactive compounds in beans, contributing to their antioxidant capacity and nutritional value^59^. The highest TFC values were obtained in the 1st stage for BF and SRF treatments, and in the 5th development stage for AS, NP, and urea treatments in terms of total flavonoid content. Similar to TPC, TFC values were low at the 3rd development stage for all treatments (Fig. 5b). Significant increases in TFC were observed from 3rd to 5th stages. The highest flavonoid content was obtained in the 5th development stage with AS treatment, and the lowest TFC was obtained in the 3rd development stage with SRF treatment. Top-dressing fertilizer treatments significantly affected flavonoid content. TFC of pods treated with SRF, NP, AS and urea was lower than that observed under BF treatment (Supplementary Table S3). Flavonoid accumulation is closely linked to nitrogen-dependent metabolic regulation, since nitrogen availability influences carbon allocation patterns and the synthesis of secondary metabolites during plant development^51,57,58^. Furthermore, flavonol glycosides, anthocyanins, and tannins (proanthocyanidins) have been reported to affect the color of the seed coat of dry beans^60^. Therefore, the increase in TFC with maturation may also be related to seed coat coloration and seed ripening.

Top-dressing fertilizer treatments significantly affected total protein content. BF and NP treatments increased total protein content consistently in bean pods from 1st to 5th stages (Fig. 5c). In contrast, total protein content decreased from 1st development stage to 2nd stage in AS, SRF and urea treatments, followed by a significant increase at the 4th and 5th stages across all treatments. The highest protein content was observed at the 5th stage, particularly for SRF treatment (Supplementary Table S3). Nitrogen is a key component of nucleic acids, amino acids, metabolic intermediates and proteins, and its availability plays a critical role in protein accumulation^61,62^. Due to the relatively low atmospheric nitrogen fixation capacity of green beans, their nitrogen fertilization requirement is higher compared to other legume species^63^. Previous studies have shown that nitrogen application increases protein content and yield in beans^64–66^. The results indicate that protein synthesis and accumulation are concentrated in the later development stages, which is consistent with enhanced nitrogen assimilation and protein biosynthesis during maturation.

Overall, phytochemical components (antioxidant capacity, phenolic compounds, flavonoids, and protein content) increased during the 4th and 5th development stages, and those top-dressing applications, especially those that have readily available nitrogen, had positive effects on these parameters. These findings contribute to identifying the optimal harvest time of fresh bean pods and linking fertilization strategies to nutritional value. When plants are exposed to environmental and biotic stresses, they may increase the production of secondary metabolites to avoid physiological and biological adverse effects. This increase can be observed in different classes of compounds, such as phenols, terpenoids, and alkaloids, depending on the type of stress^67^. In general, phenolic and flavonoid compounds are at their maximum levels initially during the development of the plants. Gradual decrease of phenolic and flavonoid compounds is associated with fruit development and maturation along with environmental conditions^68,69^. In the present study, similar trends were observed for total phenolic and flavonoid content across treatments. However, after SRF application, DPPH and CUPRAC capacity of bean pods were lower than those observed under other fertilizer treatments at all development stages. SRF is slowly released fertilizer and its presence in soil at longer periods supplements plants with their nitrogen needs. Therefore, the relatively lower antioxidant capacities observed under SRF treatment may be associated with lower accumulation of antioxidant metabolites.

### Multivariate analysis

**Fig. 6:**
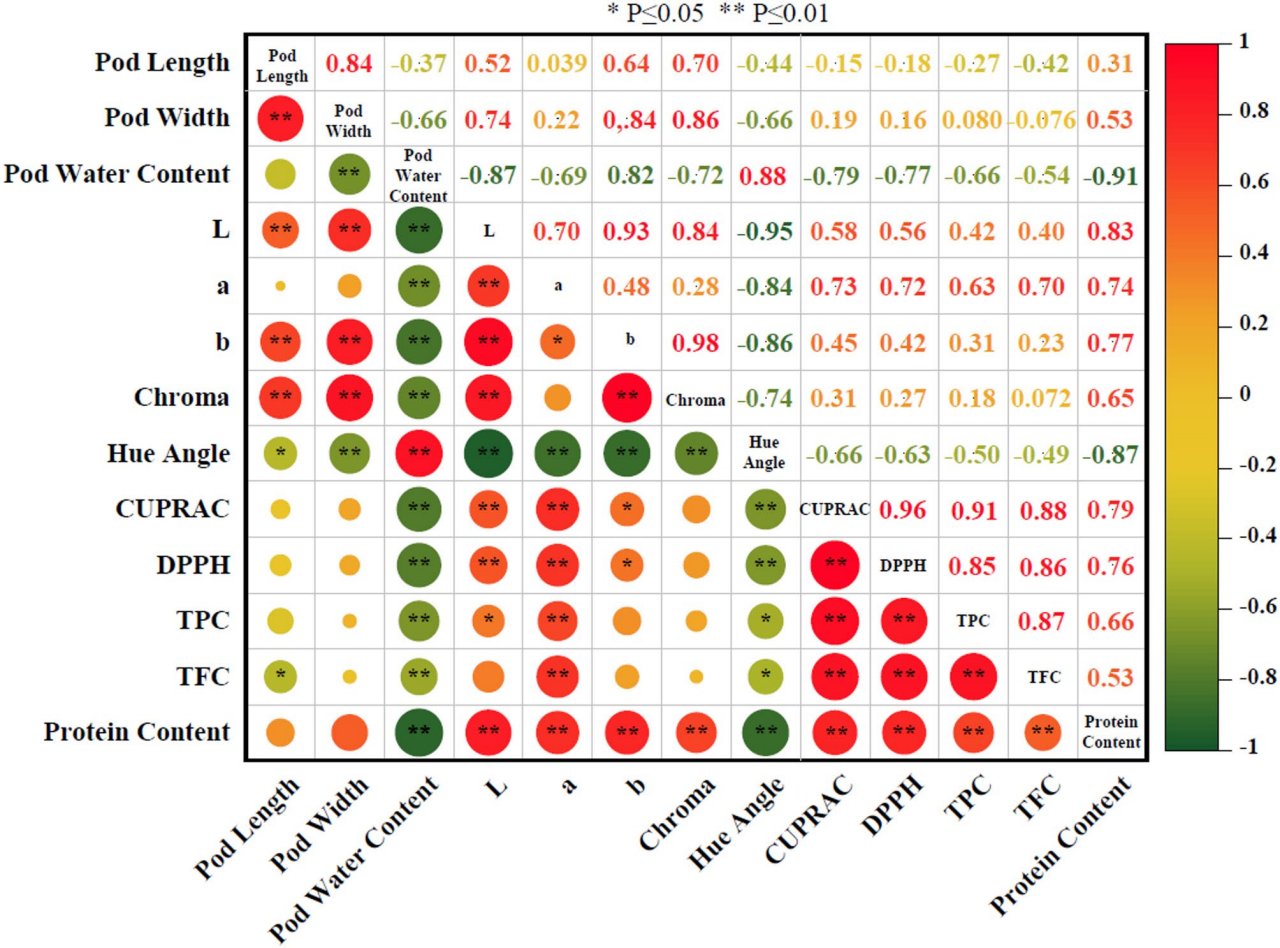
Correlation analysis of morphological characteristics, colorimetric parameters, and phytochemical components of bean pods.

The relationships between morphological characteristics (pod length, width and water content), colorimetric parameters (L*, a*, b*, chroma and hue angle), and phytochemical parameters (CUPRAC, DPPH, TPC, TFC and protein content) of bean pods were shown as a heat map using Pearson linear correlation analysis (Fig. 6). A total of 78 correlation coefficients were calculated between the parameters. Among these, 58 were statistically significant, including 36 positive and 22 negative correlations. Significant positive correlations were found between pod length and width, L*, b*, and chroma, while significant negative correlations were detected with hue angle and TFC. Significant negative correlations were found between pod water content and pod width, L*, a*, b*, chroma, CUPRAC, DPPH, TPC, TFC, and total protein content, while significant positive correlations were found with hue angle (*r* = 0.88, *p* ≤ 0.01). Hue angle showed negative correlations with the other color parameters. This reflects a transition from green to yellow coloration, associated with chlorophyll degradation and carotenoid accumulation. Previous studies have reported significant associations between color parameters and development stage and maturation^43–45^. Strong positive correlations were found between CUPRAC and DPPH, TPC, TFC, protein content (e.g., CUPRAC-DPPH, *r* = 0.96, *p* ≤ 0.01; CUPRAC-TPC, *r* = 0.91, *p* ≤ 0.01) This suggests a strong association between antioxidant capacity and phenolic and flavonoid compounds. Aquino-Bolaños et al. (2021)^70^ reported similar relationships, including negative correlations between moisture content and protein content, and between colorimetric parameters and chemical parameters in beans; and significant positive correlations between antioxidant capacity with phenolic content and color measurements. In a study conducted with 15 bean genotypes revealed that genotypes with high moisture content had low protein content (*r*=-0.81) and had negative correlation between hue angle and total protein content (*r*=-0.76)^71^.

**Fig. 7:**
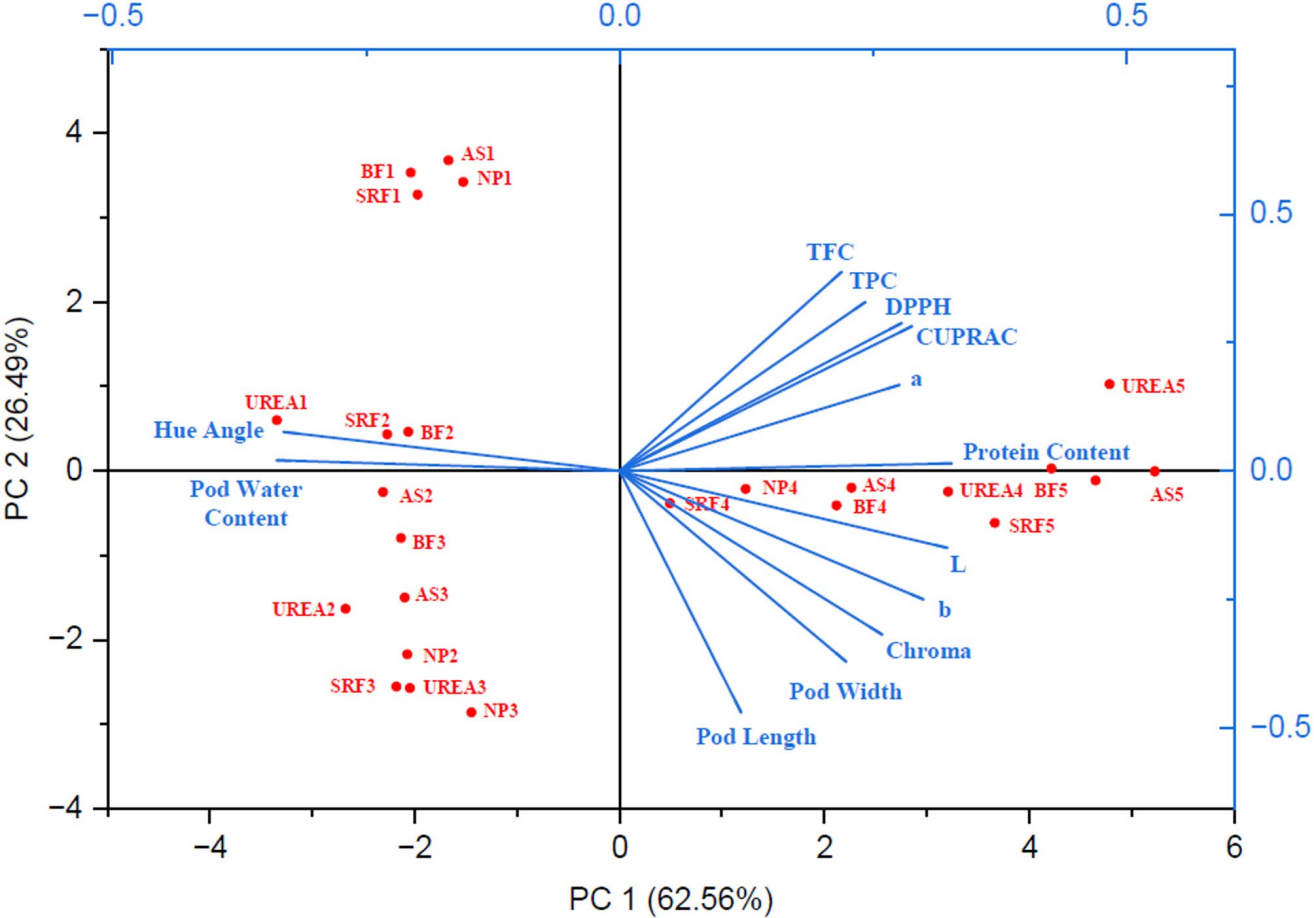
PCA analysis of morphological characteristics, colorimetric parameters, and phytochemical components of bean pods at different development stages treated with different top-dressing treatments.

The PCA results for development stages and top-dressing treatments are shown in Fig. 7. Two main components obtained from 13 variables explained 89.05% of total variance. PC1 explained the highest variance with 62.56%. Variables such as L*, b*, a*, chroma, pod width, pod length, protein content, DPPH, CUPRAC, TPC, and TFC have high positive values in this component. PC2 explained 26.49% of the variance and shared biochemical variables, such as TFC, TPC, CUPRAC, and DPPH with PC1. Regarding the effects of top-dressing treatments on pod development, the 1st, 2nd, and 3rd development stages were located in the negative range of PC1, whereas the 4th and 5th stages were positioned in the positive range. Among the examined characteristics, hue angle and pod water content were located in the negative region, while all other characteristics were positioned in the positive region of the PC1 axis. Some color parameters, such as L*, a*, and b*, may also serve as indicators of phenolic compounds and antioxidant capacity. PCA results show that these variables are grouped together and may be considered jointly in quality assessment.

## Conclusions

This study evaluated the effects of different nitrogen top-dressing treatments on the phenological, morphological, and phytochemical characteristics of bean pods during development stages. The results showed that top-dressing treatments and the development stages significantly affected pod morphology and composition. Nitrogen fertilizer treatments significantly increased the vegetative growth period by extending days to 50% flowering and pod setting time. Urea and NP treatments prolonged the growth due to rapid nitrogen release, while the slow-release SRF fertilizer provided a more balanced development course. Furthermore, top-dressing treatments increased pod length and width, particularly under NP and SRF treatments. As development progressed, green coloration decreased while yellowness increased; accordingly, L*, b*, and chroma values increased, whereas hue angle values decreased. As the harvest period progressed, antioxidant capacity (CUPRAC and DPPH), TPC, TFC and protein content increased significantly in bean pods. The highest values for these parameters were observed at later development stages (4th and 5th stages). Correlation analysis revealed strong positive associations between antioxidant activity and TPC, TFC and protein content, while negative associations were observed between pod water content and L*, a*, b*, chroma, CUPRAC, DPPH, TPC, TFC, and protein content. Since the BF treatment consisted only of basal fertilization, whereas top-dressing treatments included additional nitrogen inputs, the observed differences reflect both nitrogen source and total nitrogen availability. In addition, differences among fertilizer treatments may also be associated with variations in nitrogen release dynamics and the presence of accompanying nutrients, such as sulfur, rather than fertilizer form alone. Therefore, the results should be interpreted within the context of practical production systems rather than as a direct comparison of fertilizer forms under equal nitrogen levels. Since the study was conducted in a single growing season and at a single location, the results should also be interpreted within the specific environmental conditions of the experimental area. Further multi-year and multi-location studies are needed to confirm the consistency and broader applicability of these findings. Overall, the results indicate that both development stage and nitrogen source are key determinants of the nutritional and antioxidant quality of bean pods. Pods harvested at the 4th development stage may offer a balance between improved nutritional quality and acceptable marketability for fresh consumption. At this stage, pod elongation is completed and seed enlargement begins, while pod tenderness is generally still suitable for fresh consumption. In contrast, the 5th development stage may offer higher biochemical accumulation, but it may be less suitable for fresh consumption due to the beginning of pod maturation, increased pod firmness, and reduced tenderness. Therefore, the optimal harvest stage should be determined by balancing nutritional quality with consumer preference and market requirements. In addition, the use of more stable nitrogen sources, such as slow-release fertilizer (SRF) and ammonium sulfate (AS), may improve quality and nutritional characteristics of fresh pods.

## Supplementary Information

Below is the link to the electronic supplementary material.

Supplementary Material 1

Supplementary Material 2

## Data Availability

The data of this study is available by the corresponding author up on request.
