# Supplementary material for: Effects of nitrogen top-dressing treatments and development stages on morphological, colorimetric and biochemical characteristics of fresh bean pods (Phaseolus vulgaris L.)
Source: Sci Rep. 2026 Jun 7;16:21849. doi: 10.1038/s41598-026-57239-1 (PMC13365441; doi:10.1038/s41598-026-57239-1)
Supplement: Supplementary file 1 — Supplementary Material 1 [file 41598_2026_57239_MOESM1_ESM.docx]

**Supplementary Materials**

Table 1: Supplementary Table S1. Flowering, pod setting, and vegetation period means of beans treated with different top dressing fertilizers.

| **Top-Dressing** | **Field Observations** | | | | | **Signification** |
| --- | --- | --- | --- | --- | --- | --- |
|  | **Days to %50 Flowering** |  | **Days to %50 Pod Setting** |  | **Vegetation Period** |  |
| **BF** | 73,00 c |  | 78,00 b |  | 122,33 e | Treatment: ** |
| **AS** | 75,33 bc |  | 81,67 a |  | 134,33 c |  |
| **SRF** | 72,67 c |  | 80,00 ab |  | 129,33 d |  |
| **NP** | 77,67 ab |  | 82,33 a |  | 136,67 b |  |
| **UREA** | 79,67 a |  | 82,67 a |  | 138,67 a |  |
| **Avg.** | 75,67 |  | 80,93 |  | 132,27 |  |

Table 2: Supplementary Table S2. Morphological characteristics of pods harvested at different stages from bean plants subjected to different nitrogen top-dressing treatments.

| **Test** | **Top-Dressing** | **Pod Development Stages** | | | | | **Avg.** | **Signification** |
| --- | --- | --- | --- | --- | --- | --- | --- | --- |
|  |  | **1** | **2** | **3** | **4** | **5** |  |  |
| **Pod Length** | **BF** | 7,53c | 10,47b | 12,37a | 11,57ab | 11,53ab | 10,69b | Treatment: **  Stages: **  T x S: ** |
|  | **AS** | 7,60b | 11,1 a | 11,70a | 10,87a | 12,33a | 10,72b |  |
|  | **SRF** | 9,27b | 9,90b | 13,43a | 11,87a | 13,13a | 11,52a |  |
|  | **NP** | 8,50b | 13,17a | 12,17a | 11,87a | 12,67a | 11,67a |  |
|  | **UREA** | 9,33a | 11,47b | 13,37a | 11,50b | 11,77ab | 11,49ab |  |
|  | **Avg.** | 8,45d | 11,22c | 12,61a | 11,53bc | 12,29ab | - |  |
| **Pod Width** | **BF** | 4,91c | 7,65b | 8,58ab | 9,66a | 9,19a | 8,00ab | Treatment: **  Stages: **  T x S: ** |
|  | **AS** | 4,71d | 8,28bc | 7,43c | 9,03ab | 9,89a | 7,87ab |  |
|  | **SRF** | 4,60c | 5,59c | 8,18b | 8,98ab | 9,97a | 7,46b |  |
|  | **NP** | 5,40c | 8,01b | 9,42a | 9,55a | 8,99ab | 8,27a |  |
|  | **UREA** | 5,76c | 7,46b | 8,88a | 9,32a | 8,87a | 8,06ab |  |
|  | **Avg.** | 5,08d | 7,40c | 8,50b | 9,31a | 9,38a | - |  |
| **Pod Water Content** | **BF** | 90,88a | 91,25a | 89,09b | 67,83c | 66,18d | 81,05a | Treatment: **  Stages: **  T x S: ** |
|  | **AS** | 90,34a | 91,19a | 90,70a | 66,43b | 62,42c | 80,22b |  |
|  | **SRF** | 90,75a | 90,31a | 88,37b | 67,53c | 63,13c | 80,02bc |  |
|  | **NP** | 89,42b | 91,44a | 90,70a | 69,65c | 56,94d | 79,63c |  |
|  | **UREA** | 91,39a | 91,30a | 91,10a | 66,58b | 61,69c | 80,41b |  |
|  | **Avg.** | 90,56b | 91,10a | 89,99c | 67,61d | 62,07e | - |  |

Table 3: Supplementary Table S3. Biochemical characteristics pods harvested at different stages from bean plants subjected to different nitrogen top-dressing treatments.

| **Test** | **Top-Dressing** | **Pod Development Stages** | | | | | **Avg.** | **Signification** |
| --- | --- | --- | --- | --- | --- | --- | --- | --- |
|  |  | **1** | **2** | **3** | **4** | **5** |  |  |
| **CUPRAC** | **BF** | 4,48b | 2,74c | 2,85c | 4,49b | 5,42a | 3,40bc | Treatment: **  Stages: **  T x S: ** |
|  | **AS** | 4,77b | 3,05c | 2,24d | 4,68b | 6,09a | 4,17ab |  |
|  | **SRF** | 4,48b | 2,81c | 2,13d | 4,56b | 4,98a | 3,79d |  |
|  | **NP** | 5,23b | 2,34c | 2,15c | 5,39b | 5,81a | 4,18a |  |
|  | **UREA** | 2,70c | 2,17d | 2,09d | 5,70b | 6,62a | 3,86cd |  |
|  | **Avg.** | 4,33c | 2,62d | 2,29e | 4,96b | 5,78a | - |  |
| **DPPH** | **BF** | 2,20b | 0,75d | 1,30c | 2,40ab | 2,53a | 1,84b | Treatment: **  Stages: **  T x S: ** |
|  | **AS** | 2,37b | 1,09c | 1,01c | 2,52b | 2,97a | 1,99a |  |
|  | **SRF** | 2,19b | 1,36c | 0,58d | 2,23b | 2,67a | 1,81b |  |
|  | **NP** | 2,62a | 0,78c | 0,90c | 2,29b | 2,59a | 1,84b |  |
|  | **UREA** | 0,87c | 0,59d | 0,97c | 2,54b | 2,89a | 1,57c |  |
|  | **Avg.** | 2,05c | 0,91d | 0,95d | 2,40b | 2,73a | - |  |
| **TPC** | **BF** | 18,46bc | 21,50a | 11,61d | 17,08c | 20,24ab | 17,78a | Treatment: **  Stages: **  T x S: ** |
|  | **AS** | 20,85ab | 12,23c | 8,60d | 18,47b | 23,47s | 16,72a |  |
|  | **SRF** | 19,83a | 12,81b | 8,39c | 17,50a | 17,75a | 15,25b |  |
|  | **NP** | 19,87b | 9,87c | 8,52c | 22,44ab | 22,59a | 16,66a |  |
|  | **UREA** | 9,73b | 8,98b | 8,52b | 22,12a | 23,46a | 14,56b |  |
|  | **Avg.** | 17,75c | 13,08d | 9,13e | 19,52b | 21,5a | - |  |
| **TFC** | **BF** | 3,16a | 2,30c | 2,13c | 2,71b | 3,05a | 2,67a | Treatment: **  Stages: **  T x S: ** |
|  | **AS** | 3,00b | 2,31c | 1,83d | 2,47c | 3,25a | 2,57a |  |
|  | **SRF** | 2,80a | 2,14c | 1,59d | 2,34bc | 2,40b | 2,26b |  |
|  | **NP** | 2,63a | 1,76c | 1,66c | 2,32b | 2,74a | 2,22b |  |
|  | **UREA** | 1,88c | 1,76c | 1,67c | 2,70b | 3,16a | 2,23b |  |
|  | **Avg.** | 2,70b | 2,05d | 1,78e | 2,51c | 2,92a | - |  |
| **Protein Content** | **BF** | 42,30c | 45,21c | 45,20c | 57,37b | 65,60a | 51,13c | Treatment: **  Stages: **  T x S: ** |
|  | **AS** | 48,18c | 39,66d | 50,00c | 62,12b | 71,14a | 54,22b |  |
|  | **SRF** | 55,37b | 50,66c | 49,34c | 55,79b | 74,53a | 57,14a |  |
|  | **NP** | 50,09c | 46,23c | 50,31c | 61,75b | 72,62a | 56,20ab |  |
|  | **UREA** | 49,00b | 41,84c | 45,32bc | 73,26a | 73,40a | 56,56a |  |
|  | **Avg.** | 48,97c | 44,72d | 48,03c | 62,06b | 71,46a | - |  |
