# Supplementary material for: Effects of nitrogen top-dressing treatments and development stages on morphological, colorimetric and biochemical characteristics of fresh bean pods (Phaseolus vulgaris L.)
Source: Sci Rep. 2026 Jun 7;16:21849. doi: 10.1038/s41598-026-57239-1 (PMC13365441; doi:10.1038/s41598-026-57239-1)
Supplement: Supplementary file 2 — Supplementary Material 2 [file 41598_2026_57239_MOESM2_ESM.docx]

**Supplementary Materials**

Table 1: Supplementary Table S1. Flowering, pod setting, and vegetation period means of beans treated with different top dressing fertilizers.

| **Top-Dressing** | **Field Observations** | | | | | **Significance** |
| --- | --- | --- | --- | --- | --- | --- |
|  | **Days to 50% Flowering** |  | **Days to 50% Pod Setting** |  | **Vegetation Period** |  |
| **BF** | 73.00 c |  | 78.00 b |  | 122.33 e | Treatment: ** |
| **AS** | 75.33 bc |  | 81.67 a |  | 134.33 c |  |
| **SRF** | 72.67 c |  | 80.00 ab |  | 129.33 d |  |
| **NP** | 77.67 ab |  | 82.33 a |  | 136.67 b |  |
| **UREA** | 79.67 a |  | 82.67 a |  | 138.67 a |  |
| **Avg.** | 75.67 |  | 80.93 |  | 132.27 |  |

**BF:** basal fertilization; **AS:** ammonium sulfate; **SRF:** slow-release fertilizer; **NP:** nitropower; **UREA:** urea

Table 2: Supplementary Table S2. Morphological characteristics of pods harvested at different stages from bean plants subjected to different nitrogen top-dressing treatments.

| **Test** | **Top-Dressing** | **Pod Development Stages** | | | | | **Avg.** | **Significance** |
| --- | --- | --- | --- | --- | --- | --- | --- | --- |
|  |  | **1** | **2** | **3** | **4** | **5** |  |  |
| **Pod Length** | **BF** | 7.53c | 10.47b | 12.37a | 11.57ab | 11.53ab | 10.69b | Treatment: **  Stages: **  T x S: ** |
|  | **AS** | 7.60b | 11.1 a | 11.70a | 10.87a | 12.33a | 10.72b |  |
|  | **SRF** | 9.27b | 9.90b | 13.43a | 11.87a | 13.13a | 11.52a |  |
|  | **NP** | 8.50b | 13.17a | 12.17a | 11.87a | 12.67a | 11.67a |  |
|  | **UREA** | 9.33a | 11.47b | 13.37a | 11.50b | 11.77ab | 11.49ab |  |
|  | **Avg.** | 8.45d | 11.22c | 12.61a | 11.53bc | 12.29ab | - |  |
| **Pod Width** | **BF** | 4.91c | 7.65b | 8.58ab | 9.66a | 9.19a | 8.00ab | Treatment: **  Stages: **  T x S: ** |
|  | **AS** | 4.71d | 8.28bc | 7.43c | 9.03ab | 9.89a | 7.87ab |  |
|  | **SRF** | 4.60c | 5.59c | 8.18b | 8.98ab | 9.97a | 7.46b |  |
|  | **NP** | 5.40c | 8.01b | 9.42a | 9.55a | 8.99ab | 8.27a |  |
|  | **UREA** | 5.76c | 7.46b | 8.88a | 9.32a | 8.87a | 8.06ab |  |
|  | **Avg.** | 5.08d | 7.40c | 8.50b | 9.31a | 9.38a | - |  |
| **Pod Water Content** | **BF** | 90.88a | 91.25a | 89.09b | 67.83c | 66.18d | 81.05a | Treatment: **  Stages: **  T x S: ** |
|  | **AS** | 90.34a | 91.19a | 90.70a | 66.43b | 62.42c | 80.22b |  |
|  | **SRF** | 90.75a | 90.31a | 88.37b | 67.53c | 63.13c | 80.02bc |  |
|  | **NP** | 89.42b | 91.44a | 90.70a | 69.65c | 56.94d | 79.63c |  |
|  | **UREA** | 91.39a | 91.30a | 91.10a | 66.58b | 61.69c | 80.41b |  |
|  | **Avg.** | 90.56b | 91.10a | 89.99c | 67.61d | 62.07e | - |  |

**BF:** basal fertilization; **AS:** ammonium sulfate; **SRF:** slow-release fertilizer; **NP:** nitropower; **UREA:** urea

Table 3: Supplementary Table S3. Biochemical characteristics pods harvested at different stages from bean plants subjected to different nitrogen top-dressing treatments.

| **Test** | **Top-Dressing** | **Pod Development Stages** | | | | | **Avg.** | **Significance** |
| --- | --- | --- | --- | --- | --- | --- | --- | --- |
|  |  | **1** | **2** | **3** | **4** | **5** |  |  |
| **CUPRAC** | **BF** | 4.48b | 2.74c | 2.85c | 4.49b | 5.42a | 3.40bc | Treatment: **  Stages: **  T x S: ** |
|  | **AS** | 4.77b | 3.05c | 2.24d | 4.68b | 6.09a | 4.17ab |  |
|  | **SRF** | 4.48b | 2.81c | 2.13d | 4.56b | 4.98a | 3.79d |  |
|  | **NP** | 5.23b | 2.34c | 2.15c | 5.39b | 5.81a | 4.18a |  |
|  | **UREA** | 2.70c | 2.17d | 2.09d | 5.70b | 6.62a | 3.86cd |  |
|  | **Avg.** | 4.33c | 2.62d | 2.29e | 4.96b | 5.78a | - |  |
| **DPPH** | **BF** | 2.20b | 0.75d | 1.30c | 2.40ab | 2.53a | 1.84b | Treatment: **  Stages: **  T x S: ** |
|  | **AS** | 2.37b | 1.09c | 1.01c | 2.52b | 2.97a | 1.99a |  |
|  | **SRF** | 2.19b | 1.36c | 0.58d | 2.23b | 2.67a | 1.81b |  |
|  | **NP** | 2.62a | 0.78c | 0.90c | 2.29b | 2.59a | 1.84b |  |
|  | **UREA** | 0.87c | 0.59d | 0.97c | 2.54b | 2.89a | 1.57c |  |
|  | **Avg.** | 2.05c | 0.91d | 0.95d | 2.40b | 2.73a | - |  |
| **TPC** | **BF** | 18.46bc | 21.50a | 11.61d | 17.08c | 20.24ab | 17.78a | Treatment: **  Stages: **  T x S: ** |
|  | **AS** | 20.85ab | 12.23c | 8.60d | 18.47b | 23.47a | 16.72a |  |
|  | **SRF** | 19.83a | 12.81b | 8.39c | 17.50a | 17.75a | 15.25b |  |
|  | **NP** | 19.87b | 9.87c | 8.52c | 22.44ab | 22.59a | 16.66a |  |
|  | **UREA** | 9.73b | 8.98b | 8.52b | 22.12a | 23.46a | 14.56b |  |
|  | **Avg.** | 17.75c | 13.08d | 9.13e | 19.52b | 21.5a | - |  |
| **TFC** | **BF** | 3.16a | 2.30c | 2.13c | 2.71b | 3.05a | 2.67a | Treatment: **  Stages: **  T x S: ** |
|  | **AS** | 3.00b | 2.31c | 1.83d | 2.47c | 3.25a | 2.57a |  |
|  | **SRF** | 2.80a | 2.14c | 1.59d | 2.34bc | 2.40b | 2.26b |  |
|  | **NP** | 2.63a | 1.76c | 1.66c | 2.32b | 2.74a | 2.22b |  |
|  | **UREA** | 1.88c | 1.76c | 1.67c | 2.70b | 3.16a | 2.23b |  |
|  | **Avg.** | 2.70b | 2.05d | 1.78e | 2.51c | 2.92a | - |  |
| **Protein Content** | **BF** | 42.30c | 45.21c | 45.20c | 57.37b | 65.60a | 51.13c | Treatment: **  Stages: **  T x S: ** |
|  | **AS** | 48.18c | 39.66d | 50.00c | 62.12b | 71.14a | 54.22b |  |
|  | **SRF** | 55.37b | 50.66c | 49.34c | 55.79b | 74.53a | 57.14a |  |
|  | **NP** | 50.09c | 46.23c | 50.31c | 61.75b | 72.62a | 56.20ab |  |
|  | **UREA** | 49.00b | 41.84c | 45.32bc | 73.26a | 73.40a | 56.56a |  |
|  | **Avg.** | 48.97c | 44.72d | 48.03c | 62.06b | 71.46a | - |  |

**BF:** basal fertilization; **AS:** ammonium sulfate; **SRF:** slow-release fertilizer; **NP:** nitropower; **UREA:** urea; **CUPRAC:** cupric reducing antioxidant capacity; **DPPH:** 2,2-diphenyl-1-picrylhydrazyl radical scavenging activity; **TPC:** total phenolic content; **TFC:** total flavonoid content. Means followed by different letters within the same row or column indicate significant differences according to Tukey’s test at p ≤ 0.05. ** indicates significance at p ≤ 0.01.
